# Genomic Features of High-Level Gentamicin-Resistant Enterococcus faecalis Strain LREF-1 from India

**DOI:** 10.1128/mra.01187-21

**Published:** 2022-05-16

**Authors:** Yogesh Mudaliar, Afrah Shenaz Ahmed, Nehaa K.P., Marquess Raj, Meera J., Elavarashi Elangovan, Kumar Perumal

**Affiliations:** a Department of Biotechnology, Sri Ramachandra Institute of Higher Education and Research, Chennai, Tamil Nadu, India; b Apollo Diagnostics, Regional Reference Laboratory, Chennai, Tamil Nadu, India; c Department of Biotechnology, Sri Ramachandra Institute of Higher Education and Research, Chennai, Tamil Nadu, India; University of Maryland School of Medicine

## Abstract

Enterococcus faecalis strain LREF-1 was isolated from a urine sample from India; it belongs to sequence type 28 (ST28), which is associated with nosocomial infections. The strain carries genes for resistance to multiple antibiotics and resistance to various drug classes, including tetracycline, erythromycin, and high-level gentamicin, along with virulence factors.

## ANNOUNCEMENT

Enterococcus faecalis is a Gram-positive, commensal organism that has been gaining resistance to multiple antibiotics, particularly high-level gentamicin resistance (HLGR), in recent years. This resistance complicates treatment with combinational therapy, and the organism is a leading cause of nosocomial infections in hospital settings in India and abroad.

The study was approved by the institutional ethics committee (IEC) for student projects at Sri Ramachandra Institute of Higher Education and Research (SRIHER) (approval number CSP/21/MAR/92/265 (Ethics committee for student’s projects) for the clinical drug-resistant organism in their study). E. faecalis isolate LREF-1 was obtained from a urine sample from a 69-year-old patient, from a diagnostic laboratory in Chennai, India. The strain was cultured on brain heart infusion (BHI) agar plates, further incubated at 37°C for 24 h, and biochemically characterized as E. faecalis, and genomic DNA was extracted by using the sodium dodecyl sulfate (SDS) method (1). Antimicrobial susceptibility testing (AST) was performed using a disc diffusion assay with a single colony of the strain, according to CLSI guidelines (2). Library preparation was carried out using the NEBNext Ultra DNA library preparation kit. Sequencing was performed using an Illumina HiSeq X system (AgriGenome Labs, Kochi, Kerala, India). The numbers of Raw reads generated were 3,585,279 (Raw file 1) and 3,585,279 (Raw file 2), with a mean length of 150 bp. The raw reads (fastq files) of the sequenced genome were subjected to preprocessing, which contained two steps. In the adapter removal step, the Illumina adapter sequences were trimmed from both of the fastq files of paired-end data. After the adapter removal, Phred quality score (Q score)-based screening of the raw data was performed with a Q score cutoff value of >30. Both of the tasks were performed by using the tool Adapter removal v2, and *de novo* assembly was performed with the Unicycler assembler v0.4.8 (3). Genome annotation was performed by PGAP v5.2 (4) and RAST v2.0 (5). Antibiotic resistance genes were determined with the Comprehensive Antibiotic Resistance Database (CARD) v3.03 (6). The taxonomic position of the strain was determined by multilocus sequence typing (MLST) v2.0, which revealed that the strain belongs to sequence type 28 (ST28). The draft genome contains 65 contigs, with 3,317,115 bp. The genome coverage was 110×, and the *N*_50_ value was 201,249 bp. The strain's genome has 3,266 total genes, including 3,124 protein-coding sequences, with a GC content of 36.9% and 256 subsystems. Plasmid replicons (repUS43 in contig 3 and rep9b in contig 28) were found using MobileElementFinder v1.0.3 (7). The result revealed the strain to be a multidrug-resistant organism carrying genes for resistance to multiple antibiotics, including *ermA*, *aac(6')-Ie-aph(20)*, *lsaA*, *tetM*, *ANT(9)*, *efrA*, and *dfrE*. The *aac(6′)-Ie-aph(20)-Ia* gene is the primary gene responsible for HLGR in India. According to the 2019 NARS-Net India Report (8), 68% of the intensive care unit isolates of *Enterococcus* spp. from urine specimens have HLGR. The presence of *ANT(9)-Ia* and *lsa* genes confers resistance to aminoglycosides and clindamycin (9), respectively.

The organism was resistant to tetracycline, doxycycline, clindamycin, rifampicin, norfloxacin, erythromycin, and high-level gentamicin. Genes encoding virulence factors, including *elrA*, *ace*, *gelE*, cytolysin, *camE*, *tpx*, sex pheromone, *hylB*, *efaAfs*, and *srtA*, were determined by PATRIC v3.6.12. The strain contains Tn*554* with five substitutions and Tn*6009* with a single substitution, which contribute to tetracycline-minocycline resistance (10).

### Data availability.

The whole-genome shotgun project has been deposited in DDBJ/ENA/GenBank under the accession number JAHZSK000000000. The version described in this paper is version JAHZSK010000000. The raw sequence reads have been deposited in DDBJ/EMBL/GenBank under the SRA accession number SRR17128799.
